# Mucosa‐associated cultivable aerobic gut bacterial microbiota among colorectal cancer patients attending at the referral hospitals of Amhara Regional State, Ethiopia

**DOI:** 10.1186/s13099-021-00415-7

**Published:** 2021-03-22

**Authors:** Yesuf Adem Siraj, Melesse Gebeyehu Biadgelign, Mensur Osman Yassin, Yohannes Zenebe Chekol

**Affiliations:** 1 Department of Medical Laboratory Sciences, School of Health Sciences, College of Medicine and Health Sciences, Bahir Dar University, P.O. Box 79, Bahir Dar, Ethiopia; 2 Department of General Surgery, School of Medicine, College of Medicine and Health Sciences, Bahir Dar University, P.O. Box 79, Bahir Dar, Ethiopia; 3 Department of Surgery, School of Medicine, College of Medicine and Health Sciences, University of Gondar, P.O.Box 196, Gondar, Ethiopia; 4 CDT-Africa, College of Health Sciences, Addis Ababa University, P.O. Box 9086, Addis Ababa, Ethiopia; 5 Biotechnology Research Institute, Bahir Dar University, P.O. Box 79, Bahir Dar, Ethiopia

**Keywords:** Gut microbiota, Culture‐based, Mucosal biopsies, Colorectal cancer, Ethiopia

## Abstract

**Background:**

Colorectal cancer (CRC) is one of the top ten causes of cancer deaths in the world. Despite an increased prevalence of colorectal cancer has been documented from developing countries, there is no any report regarding gut microbiota among colorectal cancer patients in Ethiopia. Therefore, the current study evaluated cultivable aerobic gut bacterial distributions among malignant and its adjacent normal biopsies of CRC patients.

**Methods:**

CRC patients who were under colorectal cancer resection surgery during April 2017 to February 2018 at Felege Hiwot Referral and University of Gondar Teaching Hospitals enrolled in the study. Biopsy specimens were taken from malignant and its adjacent normal-appearing tissues. Bacterial cultivation, quantification and characterization of saline washed biopsies were performed under aerobic and candle jar conditions. Differences in bacterial microbiota compositions between malignant and normal tissue biopsies were evaluated and analyzed using Microsoft excel 2010 and GraphPad Prism5 statistical software.

**Results:**

Fifteen CRC patients were participated with a mean age of 53.8 ± 10.8 years old and majorities (73.3 %) of patients were in between the age groups of 40 and 60 years old. The mean ± SD bacterial microbiota of malignant biopsies (3.2 × 10^5^ ± 1.6 × 10^5^ CFU/ml) was significantly fewer than that of adjacent normal tissue biopsies (4.0 × 10^5^ ± 2.2 × 10^5^ CFU/ml). This dysbacteriosis is positively correlated with the occurrence of CRC (p = 0.019). Proteobacteria (55.6 %), Firmicutes (33.3 %) and Fusobacteria (11.1 %) were the most frequently isolated phyla from non-malignant biopsies while only Proteobacteria (58.8 %) and Firmicutes (41.2 %) were from malignant ones. Family level differences were observed among phyla (Firmicutes and Proteobacteria) isolated from the study participants. For instance, the relative abundance of family Bacillaceae from malignant (26 %) was lower than the normal biopsies (39 %). On other hand, family Enterobacteriaceae was twice more abundant in malignant tissues (45 %) than in its matched normal tissues (23 %). Furthermore, the family Enterococcaceae (14 %) of phylum Firmicutes was solely isolated from malignant tissue biopsies.

**Conclusions:**

The overall microbial composition of normal and malignant tissues was considerably different among the study participants. Further culture independent analysis of mucosal microbiota will provide detail pictures of microbial composition differences and pathogenesis of CRC in Ethiopian settings.

**Supplementary Information:**

The online version contains supplementary material available at 10.1186/s13099-021-00415-7.

## Background

Colorectal cancer (CRC) is the fourth most common causes of cancer deaths in the world with about 900,000 deaths annually [1] next to lung cancer [2]. It accounts for approximately 10 % of cancer-related mortality in western countries [3]. Although a population based data is unavailable from Ethiopia, colorectal cancer is a major problem with significant magnitude of unresectable tumors [4]. Based on a single cancer registry data of Addis Ababa City, the Global cancer statistics center reported 4716 (7 %) new CRC cases in 2018 [2] which makes CRC ranked at the third of cancer cases in Ethiopia.

Various non-modifiable and environmental factors are casually associated with the incidence of colorectal cancer. Age and hereditary factors are among non-modifiable factors that cannot be controlled by an individual while environmental factors including dietary change, urban residence, smoking habit, heavy alcohol consumption, and physical inactivity and obesity are considered as modifiable factors [5].

Inflammatory bowel diseases (ulcerative colitis and Crohn’s disease) are keenly associated with the development of colorectal cancer in which various immune regulatory pathways have been identified in ulcerative colitis associated CRC [5, 6]. Among these, chronic intestinal inflammation, defective mucosal barrier and host-microbe dynamics promote tumorigenesis of colorectal regions of gastrointestinal tract [6]. Moreover, microbial-induced chronic colitis drives the progression of adenoma to invasive carcinoma [7].

In spite of microbial composition of the human intestine is obviously correlated to the health conditions, human gut microbiota have emerged as a major environmental factor that modulate the risk of colorectal cancer. Dysbiosis of gut microbiota [8, 9] is now assumed to be an underlying factor in the development of colorectal cancer. Currently, several researches are trying to associate the change in the composition of human intestinal microbiota with colorectal cancer occurrence. However, most studies might not show strong association due to different constraints including use of non-intestinal biopsy investigations and convenience of specimen [10]. Mucosa-associated microbiota potentially affects CRC risk primarily through direct interaction with the host [11] and its significantly differed organization in CRC patients and healthy individuals [12].

Granting an increase in prevalence of colorectal cancer has been documented from developing countries [2, 13], reports on gut microbiota in relation to colorectal cancer are not yet issued particularly in Ethiopia. Phenotypic, genotypic and toxin gene analyses of gut microbiota composition have not yet been done among colorectal cancer patients in the study area and in Ethiopia at large. Therefore, this study is aimed at determining the microbial distribution and characterizing cultivable aerobic gut mucosal associated bacteriobiota among cancerous and adjacent apparently normal tissues of colorectal cancer patients.

## Result

Fifteen colorectal cancer patients were recruited from two referral hospitals: Felege Hiwot Referral Hospital (n = 8) and University of Gondar Teaching Hospital (n = 7). Nine (60 %) were males with a male to female ratio of 1.5:1. The cumulative mean age ± SD of the study participants were 53.8 ± 10.8 years with a range of 38 and 79 years old. Eleven (73.3 %) of the study participants were between the age groups of 38 and 60 years of the first two quartiles while elders with ≥ 60 years old were only 4 (26.7 %) (Table 1).

**Table 1:** Socio-demographic characteristics of study participants with colorectal cancer

	1st Quartile	2nd Quartile	3rd Quartile	Total
	N (%)	N (%)	N (%)	N (%)
	4 (26.7)	7 (46.6)	4 (26.7)	15 (100)
Age (in years)				
Mean ± SD	40.8 ± 3.6	54.6 ± 4.7	65.5 ± 9.1	53.8 ± 10.8
Median	39.5	56	61.5	56
Minimum	38	48	60	38
Maximum	46	59	79	79
Sex				
Male N (%)	3 (75)	4 (57)	2 (50)	9 (60)
Female N (%)	1 (25)	3 (43)	2 (50)	6 (40)
Male:female ratio	1.5:1			

**Fig. 1:**
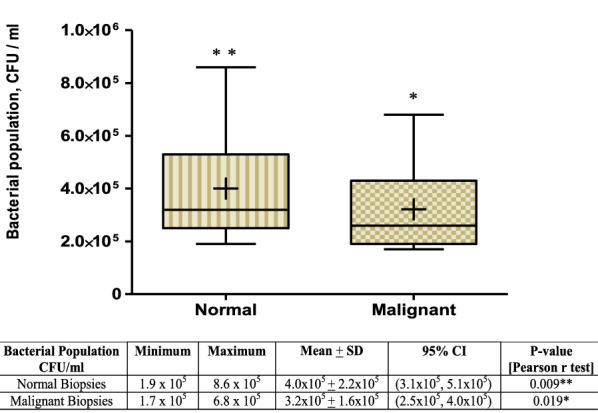
Box-Whisker plot of bacterial microbiota abundance in normal and malignant tissue biopsies of CRC patients. The plot shows median values, means (+ sign in boxes), interquartile ranges (IQR) (boxes) and 1.5 × IQR (whiskers). Bacterial population isolated from paired biopsies of CRC patients was significantly associated with the occurrence of tumor at (*p < 0.05) or being normal tissue at (**p < 0.01)

The overall abundance of cultivable aerobic bacteria was recovered from triplicate culture plates and compared with types of biopsies. The mean ± SD population of aerobic bacteria cultivated from normal-featuring biopsies was approximately 4.0 × 10^5^ ± 2.2 × 10^5^ CFU/ml while it was 3.2 × 10^5^ ± 1.6 × 10^5^ CFU/ml from malignant tissues. According to the Pearson r test, significant correlation was observed between a reduced bacterial microbiota (dysbacteriosis) of washed malignant tissue suspensions and the occurrence of colorectal cancer (p = 0.019, Pearson r = 0.596, 95 % CI = 0.120–0.849) (Fig. 1).

As the Box-Whiskers appearance indicates, the mean bacterial population of malignant was significantly different from the adjacent normal tissues biopsies at p < 0.05 (Fig. 1). The relative abundance of bacteria at family or genus level in each cancerous specimen was much smaller compared to the other equivalent normal tissue biopsies. The upper range value of bacterial abundance of malignant tissues [6.8 × 10^5^ CFU/ml] was reduced at a minimum of 2.0 × 10^5^ CFU/ml of washed biopsy suspension from its matched normal tissue biopsies count [8.6 × 10^5^ CFU/ml]. Similarly, the lower range value of washed malignant tissue biopsies [1.7 × 10^5^ CFU/ml] was also 2.0 × 10^4^ CFU/ml fewer than its equivalent counts of adjacent normal tissues [1.9 × 10^5^ CFU/ml] (Fig. 1).

**Fig. 2:**
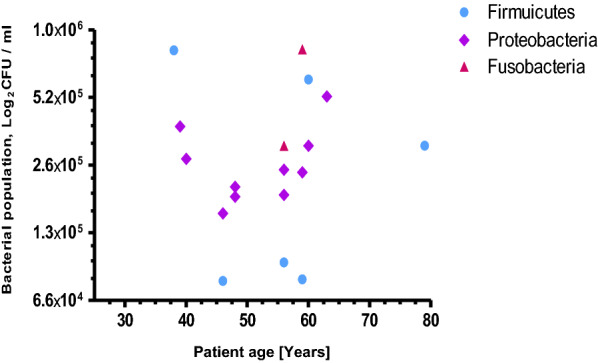
Age-specific bacterial phylum distribution isolated from normal tissue biopsies of CRC patients

**Fig. 3:**
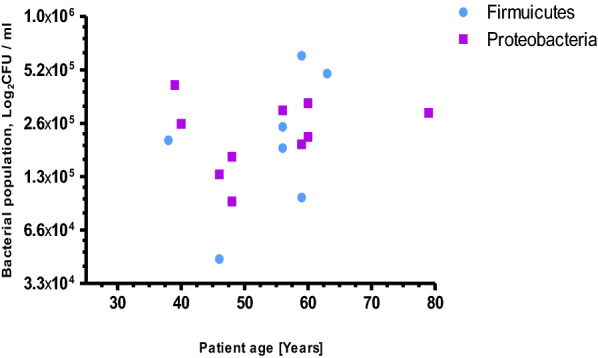
Age-specific bacterial phylum distribution isolated from malignant tissue of biopsies of CRC patients

Comparing the mucosal microbiota of malignant niche to its matched adjacent normal tissues indicated varied bacterial compositions over those two groups of samples of CRC patients. Three bacterial phyla; Proteobacteria (55.6 %), Firmicutes (33.3 %) and Fusobacteria (11.1 %) were over represented in non-malignant tissues of CRC patients (Fig. 2) while only two phyla; Firmicutes (41.2 %) and Proteobacteria (58.8 %) were recovered from malignant biopsies of CRC patients (Fig. 3). In addition, more bacterial diversity has been observed from apparently healthy tissue specimens group than its equivalent particularly among the age groups of 55 to 65 years (Figs. 2, 3).

**Fig. 4:**
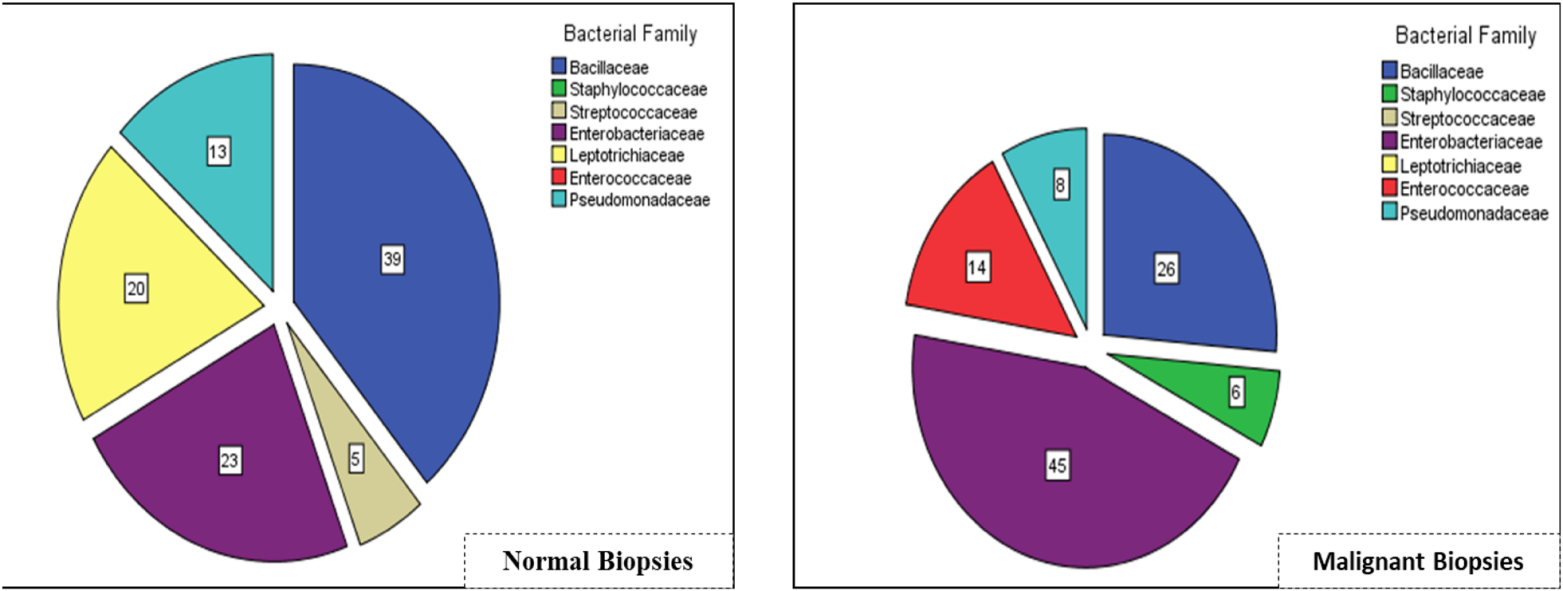
Bacterial family distribution in Normal and Malignant biopsies of CRC patients. Numbers are in percentage of the total family coverage

Though most members of phylum Fusobacteria are obligate anaerobic bacteria, a genus *Streptobacillus* (Fig. 4) under a family Leptotrichiaceae (20 %) with a microaerophilic nature was recovered only from the normal tissue biopsies using CO_2_ enriched cultivation. Phyla Firmicutes and Proteobacteria recovered from both groups of tissues showed no difference while family level differences between biopsy groups were observed. The relative abundance of family Bacillaceae isolated from non-malignant tissue biopsies was at (39 %) of the total isolated bacterial families while it was much lower proportion (26 %) from malignant tissue biopsies. On the other hand, the relative abundance of family Enterobacteriaceae (45 %) isolated from malignant tissue was twice higher than from the matched control biopsies (23 %). Furthermore, the family Enterococcaceae (14 %) was isolated only from malignant biopsies of CRC patients (Fig. 4).

## Discussion

The variability of microbial population of gastrointestinal tract is currently correlated to the occurrence of different disorders including colorectal cancer. Though several recent advanced researches in developed countries use genomic and metagenomic approaches to characterize microbial cells in feacal or mucosal specimens, there is no any published data related to the overall microbiota profile of mucosal or feacal specimens of CRC patients in Ethiopia. Therefore, the current study was aimed at determining the distribution of at least cultivable aerobic bacterial microbiota of cancerous and normal-featuring tissues of CRC patients.

The dysbiosis of bacterial microbiota abundance and distribution in malignant tissues from adjacent normal biopsies is currently become an indicative in the diagnosis and prognosis of CRC patients. These alterations are also demonstrated in our study by the presence of abundant bacterial microbiota in normal biopsies [x̄=4.0 × 10^5^ CFU/ml] while much smaller bacterial population [approximately 2.0 × 10^5^ CFU/ml less] from malignant tissue biopsies of CRC patients (Fig. 1).

In this study, we found higher abundance of bacterial composition of phyla; Proteobacteria (55.6 %), Firmicutes (33.3 %) and Fusobacteria (11.1 %) in normal biopsies of CRC patients (Fig. 2). However, it is much different from a study reported by Eckburg et al. [14], in which 90 % of bacterial composition of normal luminal microbiota belongs to the phyla; Firmicutes and Bacteriodes, the remaining minor constituents were Proteobacteria and Actinobacteria. On the other hand, a review study by Villeger et al. [15] presented a clear increase in both Proteobacteria and Bacteroidetes in tissues from patients with colorectal adenoma compared with tissues from healthy volunteers. Among members of phylum Fusobacteria [16], only a genus *Streptobacillus* in the family Leptotrichiaceae was isolated from normal tissues of CRC patients (Fig. 2). It could be due to the alternative method we employed, candle jar for fastidious bacterial cultivation, probably supported the growth of microaerophilic bacteria. Other most fusobacterial members strictly require anaerobic environment to grow [17] and are associated greatly with cancer tissues than in normal tissues [18]. Despite the genera Bacteriodes [19], Leptotrichia species [20, 21] and Fusobacteria [19, 22] were the most frequently identified and reported bacteria from malignant tissues of colorectal cancer, our study didn’t showed any above mentioned species while we employed candle jar cultivation.

According to the author Lau et al. [23], *Streptobacillus hongkongensis* is a novel bacterial species that permanently found in human oropharynx and there might be more other *Streptobacillus* species probably also residing in human oropharynx. This genus might get easy access to the lumen of the colorectal regions [24].

The microbial abundance of family Bacillaceae in malignant biopsies (26 %) was lower than the abundance in non-malignant tissues (39 %) while the family Enterobacteriaceae, a member of phylum Proteobacteria [25] was over-represented (45 %) from malignant group of tissue (Fig. 4). This observation could be supported by the fact that family Enterobacteriaceae is considerably a member of the carcinogenic bacteria that constitute Lipopolysaccharides (LPS), d-Lactate and other bacterial components which positively correlated with the incidence and progression of inflammatory bowel diseases (IBD) as well as colorectal cancer [26–28].

More recently, studies [15, 29] indicated a drastic difference in the microbial composition has been observed in the mucosa of colitis-associated CRC patients, with an increase in the family Enterobacteriaceae compared with the mucosa of sporadic CRC patients. For instance, colibactin-producing *E. coli* were more frequently identified in microsatellite stable CRC and higher colonization by negative-colibactin *E. coli* bacteria were detected in patients with microsatellite instability CRC [15].

Our study also revealed that significant abundance of family Enterococcaceae was identified only from malignant biopsies (Fig. 4). This finding supports previously reported evidences that patients with ulcerative colitis and Crohn’s disease have larger members of family Enterococcaceae than healthy controls [30–32]. Furthermore, genus Enterococcus is among certain bacterial species that has been identified to play a key role in the incidence and development of colorectal cancer [15, 29]. In this genus, *Enterococcus faecalis* can even trigger macrophages and other immune cells to produce procarcinogenic enzymes capable of damaging target cell DNA that contribute to CRC carcinogenesis [33, 34].

The imbalance of these bacteria and their gene products [35, 36] that underlies mucosal surface of intestinal microvilli would facilitate the replication of opportunistic pathogens which might have direct contribution in the onset and progression of severe gastrointestinal inflammation leading to colorectal cancer. Hence, these findings could be a base of future investigations focusing on potential pro-oncogenic pathogens of gastrointestinal cancers in the study area.

### Strengths

The current study used intact biopsies of both malignant and adjacent normal appearing tissues of study participants where most faecal specimen microbiome studies might not show typical characteristics of adherent gut microbiota of the colorectal cancer patients.

### Limitations

Colonoscopy is an invasive procedure to get intestinal tissue biopsies from healthy individuals; consequently we didn’t include apparently healthy individuals as a control group. Furthermore, our study employed a culture-based aerobic cultivation, huge segment of mucosal-associated microbiota such as obligate anaerobes, fungal agents and uncultivable microbes were not addressed. Microbial distributions in relation to anatomic positions of colorectal biopsies, cancer stage, anticancer or antibiotic use, comorbid diseases and long term dietary habit were not considered. However, with these limitations, the study will provide base line information for future development of culture independent studies of gut microbiota in the study area.

## Conclusions

Findings presented in the current study suggested a relative abundance and distributions of cultivable aerobic bacterial microbiota of malignant tissues were significantly different from its adjacent normal tissue biopsies. Our study also showed that families of Enterobacteriaceae and Enterococcaceae were the most frequently recovered bacterial family from malignant tissues while detail considerations of these bacteria in the initiation and progression of colorectal cancer remains unclear. Therefore, large scale and deep metagenomic analysis of gut microbiota differences in Ethiopian population play key roles in the future development of advanced diagnostic, prognostic and therapeutic strategies of colorectal cancer patients.

## Methods

### Patient recruitment and mucosal biopsy

At Gasteroenterology and Digestive Clinics of Felege Hiwot Referral and University of Gondar Teaching Hospitals, 15 confirmed CRC patients who underwent surgical resections of cancerous tissues were enrolled in the study during the study period of April, 2017 to February, 2018. Patients who were confirmed solely for colorectal cancer and appointed for open colorectal surgery were included in the study. These patients were instructed for bowel preparation before surgery carried out. However, patients who had been administered with antibiotics in the last two weeks prior to surgery, who exhibited either metastases or other cancers (liver, pancreatic and lung cancers) and who presented insufficient tissue biopsies were excluded from the study. Informed consent from each study participant was obtained and information was kept confidential. Two biopsies (with 5–7 × 5–7 mm dimensions) were collected from malignant and adjacent normal-appearing tissues of the colorectal lumen of CRC patients during open resection surgery. Each biopsy specimen was aseptically collected using sterile falcon tube containing sterile normal saline and immediately processed in the bacteriology laboratory. Saline washed biopsy suspensions were used for aerobic cultivation. Biopsy specimens were preserved at 4 °C where delayed analysis was unavoidable. Findings were analyzed and interpreted accordingly using statistical software.

### Bacterial count and identification

All collected biopsies were intensively washed with 5 ml of normal saline. Twenty µl suspension of each saline-washed specimen was suspended on to each three plates of meat peptone agar. MacConkey agar, a selective media, was also employed to isolate common pathogenic bacteria like *Salmonella* and *Shigella* species. Colony forming unit (CFU) count, morphological characteristics of bacterial isolates at average logarithmic growth phase and identification of bacterial species using a series of biochemical tests were aseptically performed. Sterility and performance of the prepared media were checked by parallel inoculation of locally available control strains of American Type Culture Collection: *S. aureus* (ATCC^®^-25,923), *P. aeruginosa* (ATCC^®^-27,853) and *E. coli* (ATCC^®^-25,922).

### Statistical analysis

Before we performed statistical analysis, the data were transformed into a natural logarithm (ln) of bacterial populations of malignant and adjacent normal tissues. Hence, positive skewness of our data was changed from 1.386 to 0.810 with acceptable normal distribution (Additional file 1: S1 Figure, S2 Figure). Statistical data analysis and plotting were performed using Microsoft excel 2010, GraphPad Prism5 or SPSS version 20 software accordingly. Pearson r test, mean and standard deviation were employed. Statistically significant level was considered at p ≤ 0.05.

## Supplementary Information

**Additional file 1: Figure S1.** Normal distribution of bacterial population isolated from adjacent normal biopsies of colorectal cancer patients. Original bacterial population data were transformed using a natural logarithm function (ln) and Pearson r correlation test was performed using transformed data. **Figure S2.** Normal distribution of bacterial population isolated from malignant biopsies of colorectal cancer patients. Original bacterial population data were transformed using a natural logarithm function (ln) and Pearson r correlation test was performed using transformed data.

## Data Availability

The datasets collected and analyzed in the present study are available with the first author up on request.

## Abbreviations

CAC: Colitis-associated cancer
CFU: Colony forming unit
CRC: Colorectal cancer
IBD: Inflammatory bowel disease
IQR: Interquartile range
IRB: Institutional Review Board
