# Supplementary material for: Mucosa‐associated cultivable aerobic gut bacterial microbiota among colorectal cancer patients attending at the referral hospitals of Amhara Regional State, Ethiopia
Source: Gut Pathog. 2021 Mar 22;13:19. doi: 10.1186/s13099-021-00415-7 (PMC7983201; doi:10.1186/s13099-021-00415-7)
Supplement: Supplementary file 1 — Additional file 1: Figure S1. Normal distribution of bacterial population isolated from adjacent normal biopsies of colorectal cancer patients. Original bacterial population data were transformed using a natural logarithm function (ln) and Pearson r correlation test was performed using transformed data. Figure S2. Normal distribution of bacterial population isolated from malignant biopsies of colorectal cancer patients. Original bacterial population data were transformed using a natural logarithm function (ln) and Pearson r correlation test was performed using transformed data. [file 13099_2021_415_MOESM1_ESM.pptx]

## Slide 1
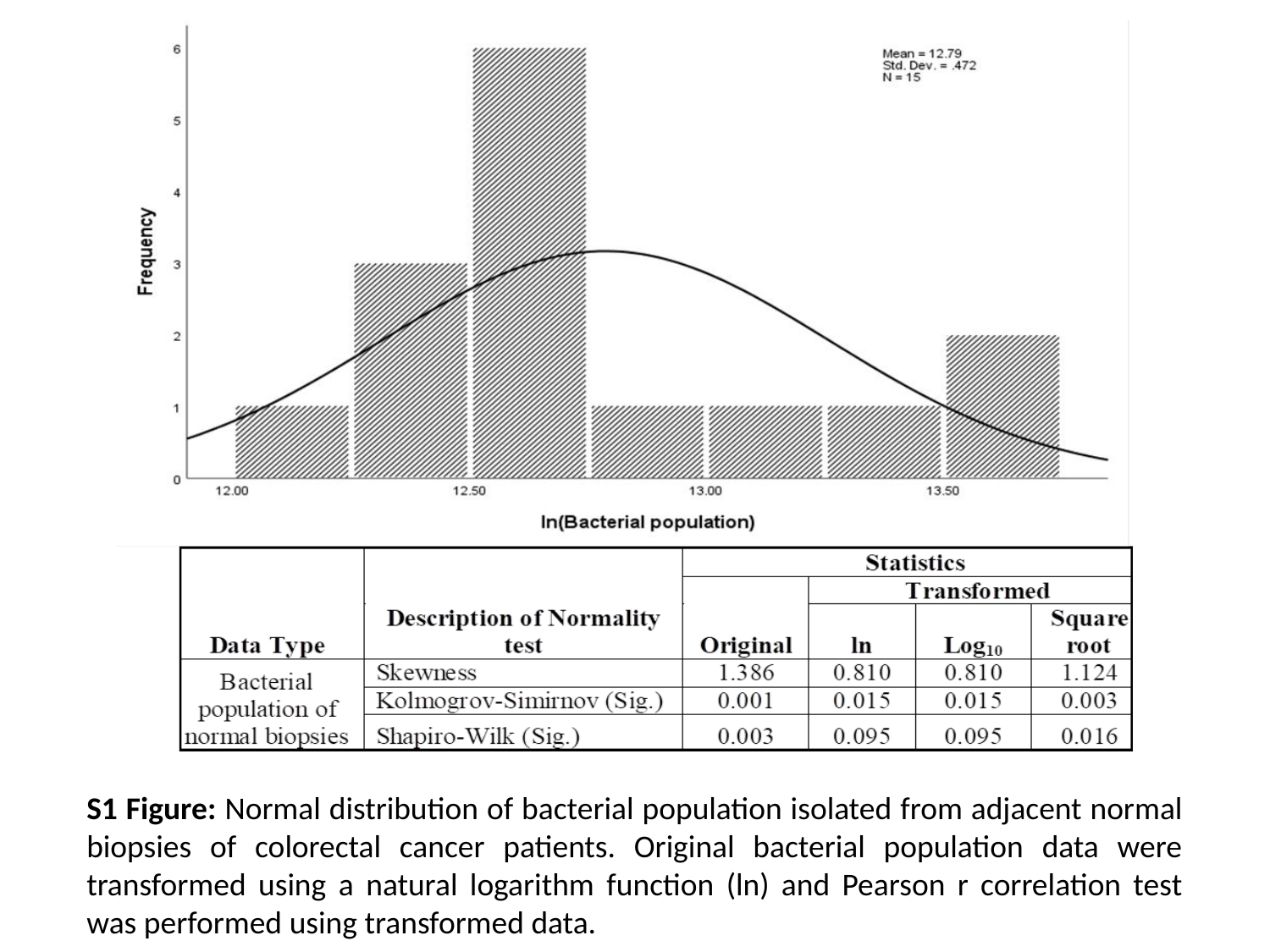

S1 Figure: Normal distribution of bacterial population isolated from adjacent normal biopsies of colorectal cancer patients. Original bacterial population data were transformed using a natural logarithm function (ln) and Pearson r correlation test was performed using transformed data.

## Slide 2
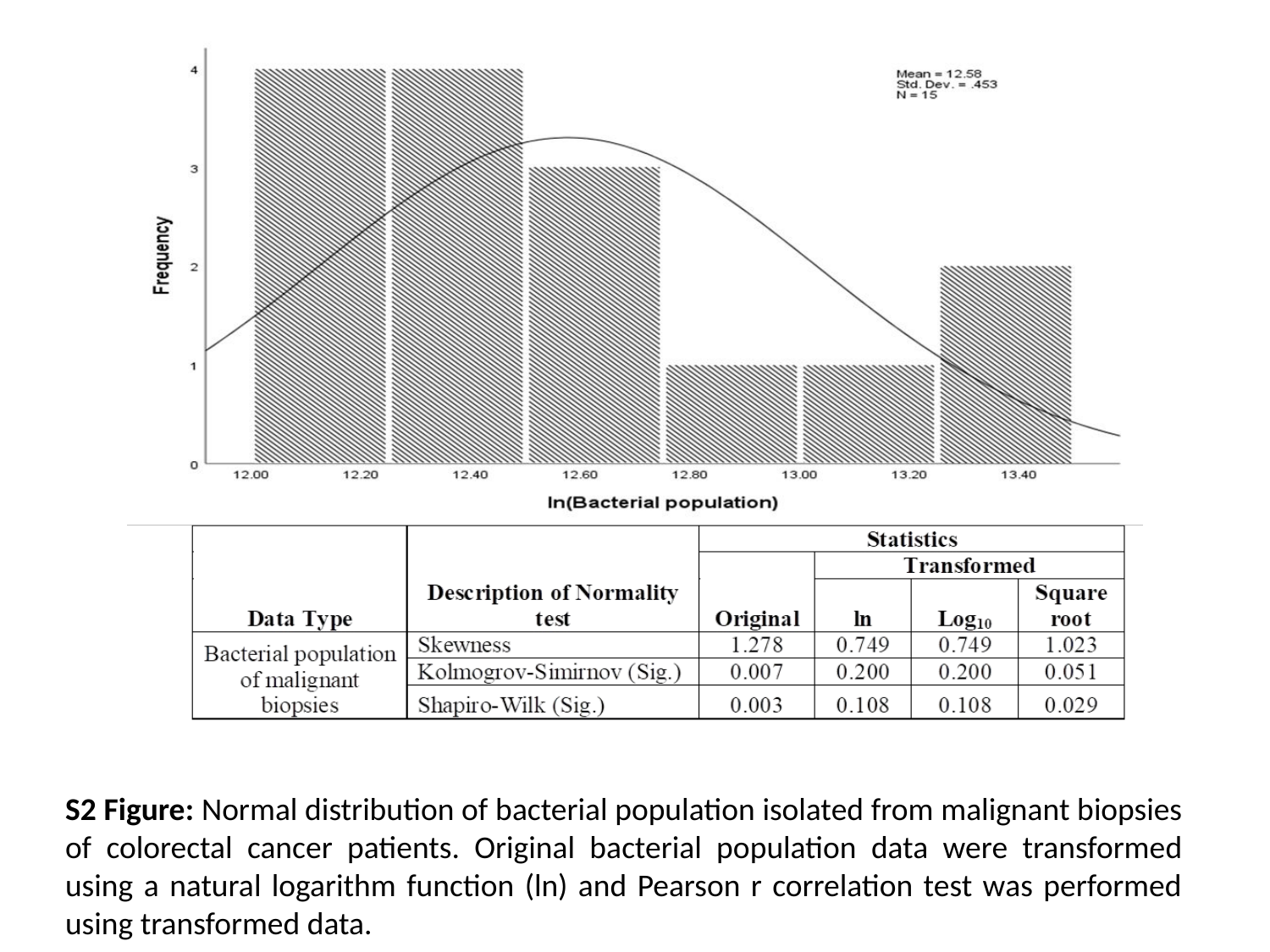

S2 Figure: Normal distribution of bacterial population isolated from malignant biopsies of colorectal cancer patients. Original bacterial population data were transformed using a natural logarithm function (ln) and Pearson r correlation test was performed using transformed data.
